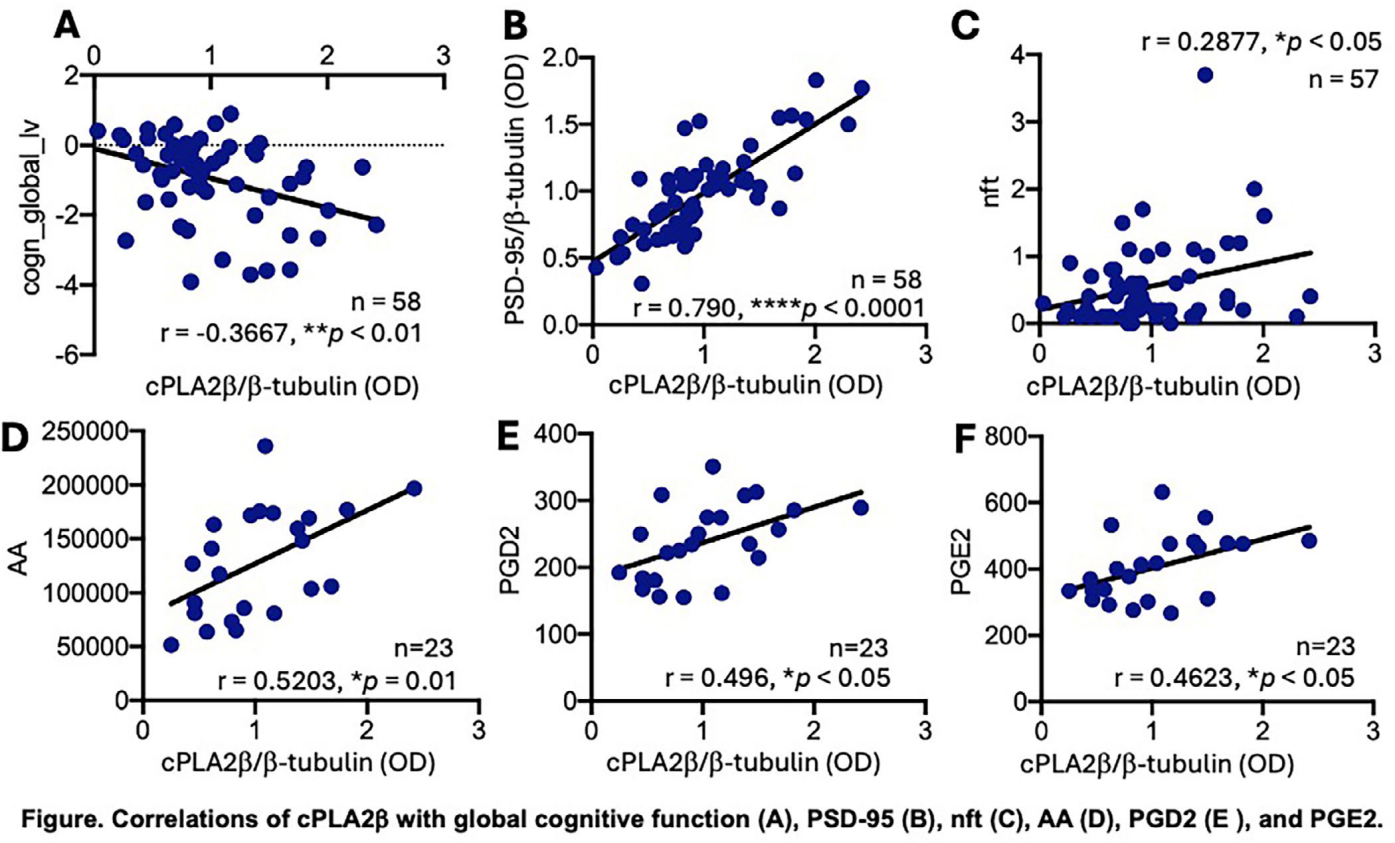# Novel mechanisms of cytosolic phospholipase A2 beta in neurodegeneration and cognitive impairments of Alzheimer’s disease

**DOI:** 10.1002/alz.092113

**Published:** 2025-01-03

**Authors:** Qiulan Ma, Brandon Ebright, Jing Li, Dante Dikeman, Ashley Sanchez, Jasmin Galvan, Daniel Badie, Boyang Li, Shaowei Wang, Bilal Ersen Kerman, Debra Hawes, Kyle Hurth, David A. Bennett, Zoe Arvanitakis, Stan G Louie, Hussein N Yassine

**Affiliations:** ^1^ University of Southern California, Los Angeles, CA USA; ^2^ USC School of Pharmacy, Los Angeles, CA USA; ^3^ USC Keck School of Medicine, Los Angeles, CA USA; ^4^ Rush University, Chicago, IL USA; ^5^ Rush Alzheimer’s Disease Center, Rush University Medical Center, Chicago, IL USA

## Abstract

**Background:**

Synaptic loss predicts cognitive decline in Alzheimer’s disease (AD). However, the critical disease modifying molecular mechanisms of synaptic failure remain elusive. Animal studies implicate the increased activation of cytosolic phospholipase (cPLA2) activation in synaptic loss and neuroinflammation. cPLA2 activation in human brain synapses have not yet been demonstrated. cPLA2 preferentially catalyzes the conversion of arachidonic acid (AA) to generate eicosanoids: lipid mediators of inflammation. Synaptosomes represent an excellent ex vivo model to study synaptic function in humans. Both PSD‐95 and CamKII are synaptic markers that play essential role in learning and memory. In this study, we sought to determine whether cPLA2β is associated with synaptic markers, AD pathology and cognition in synaptosomes derived from human brain tissues.

**Method:**

cPLA2β expression was analyzed in synaptosomes isolated from midfrontal cortex of postmortem brains in participants with no cognitive impairment (NCI, n = 20), mild cognitive impairment (MCI, n = 14) and AD (n = 19) from the clinically and pathologically well‐characterized Religious Orders Study (ROS). Experimental methods included Western blotting, immunofluorescence staining, and liquid chromatography‐tandem mass spectrometry to measure AA and its metabolites.

**Result:**

cPLA2β was increased in synaptosomes/synapses of both MCI (*p < 0.05) and AD (***p < 0.001) compared to NCI. cPLA2β was inversely corelated with global cognitive function (r = ‐0.3667, **p < 0.01), and positively correlated with neurofibrillary tangles (r = 0.2877, *p < 0.05). Interestingly, cPLA2β was highly positively correlated with postsynaptic proteins of PSD‐95 (r = 0.7901, ****p < 0.0001) and calcium/calmodulin dependent protein kinase II (CaMKII, r = 0.2825, *p<0.05). Immunofluorescence staining revealed the colocalization of cPLA2β with phospho‐tau (AT8), synaptic PSD‐95 and CaMKII in both AD and NCI brains. cPLA2β stained degenerative excitatory neurons. On a functional level, cPLA2b was positively correlated with AA (r = 0.5203, *p = 0.01) and its inflammatory metabolites of PGD2 (r = 0.496, *p<0.05) and PGE2 (r = 0.4623, *p<0.05).

**Conclusion:**

This is the first report revealing increased expression of cPLA2β in synaptosomes from brains with MCI and AD compared with controls. cPLA2β is associated with tau pathology, AA and its metabolites, synaptic and cognitive performance. Whether reducing cPLA2b reverses AD pathology merits further investigation.